# Construction of a Dual Protease Column, Subzero (-30 °C) Chromatography System and Multi-channel Precision Temperature Controller for Hydrogen-Deuterium Exchange Mass Spectrometry

**DOI:** 10.6028/jres.125.025

**Published:** 2020-08-12

**Authors:** Jeffrey W. Hudgens

**Affiliations:** 1 National Institute of Standards and Technology, Bioprocess Measurement Group, Biomolecular Measurement Division, Gaithersburg, MD 20899, USA; 2 Institute for Bioscience and Biotechnology Research, 9600 Gudelsky Drive, Rockville, MD 20850, USA

**Keywords:** chromatography, hydrogen-deuterium exchange, mass spectrometry, precision, peptide, protein, proteolysis, proteomics, reference material, temperature control

## Abstract

This tutorial provides mechanical drawings, electrical schematics, parts lists, stereolithography (STL) files for producing three-dimensional (3D)-printed parts, initial graphics exchange specification (IGS) files for automated machining, and instructions necessary for construction of a dual protease column, subzero, liquid chromatography system for hydrogen-deuterium exchange mass spectrometry (HDX-MS). Electro-mechanical schematics for construction of two multi-zone temperature controllers that regulate to ±0.05 oC are also included in this tutorial.

## 1 Introduction

This tutorial provides mechanical drawings, electrical schematics, parts lists, STL files for 3D-printed parts, EPS files for automated machining, and instructions necessary for construction of a dual protease column, subzero, liquid chromatography (LC) system. This document also presents electrical schematics for two different six-zone temperature controllers that can regulate each zone to ±0.05 ^o^C.^1^1 This tutorial lists a temperature regulation specification of ±0.05 ^o^C; however, as is annotated on the schematics in the NIST Public Data Repository, one temperature controller design regulates to ±0.10 ^o^C, and a second design regulates to ±0.05 ^o^C.

Hydrogen-deuterium exchange mass spectrometry (HDX-MS) is employed increasingly during the development and registration of bio-pharmaceuticals [1-3]. New applications, particularly those involving quality control and biosimilarity evaluations, will demand deuterium uptake measurements of improved precision with minimized H for D back-exchange and isotopic bias [4-6]. Existing HDX-MS analysis systems that digest samples and use liquid chromatography to separate peptides at pH 2.5 and 0 ^o^C can lose > 30% of the deuterium marker within 20 minutes of sample injection [7-18]. Isotopic bias from sample carryover and trapped protein particulates is also a known problem [6, 19, 20].

The system (Fig. 1) presented here performs proteolysis on samples at 0 ^o^C and chromatographic separations at -30 ^o^C, essentially eliminating back exchange during chromatographic separations. Total back-exchange is ≤ 10%, as governed by the sample preparation process. Subzero operation is enabled by mixing ethylene glycol (ETG) with the proteolytic peptides to make a 45% by volume (v) ETG solution. Back-flushing the proteolytic and chromatographic columns essentially eliminates carryover.

**Fig. 1:** Features of the subzero **(**-30 ^o^C) protease and chromatography system. (A) HDX-MS system clamped to a commercial, linear robotic rail and a list of improvements over the current art. (B) View of the components of the fluidic circuit within the HDX-MS dual protease column, subzero chromatography system. **Table:** 

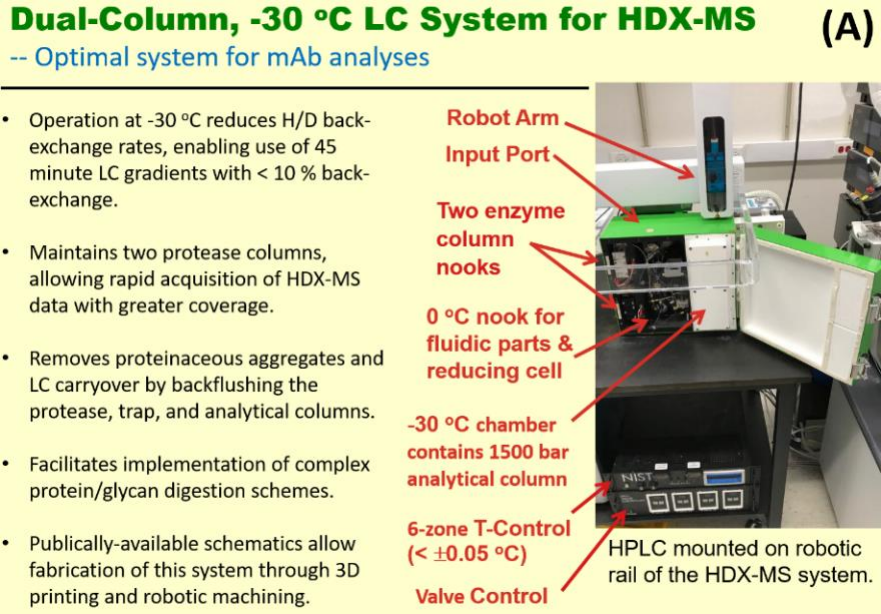
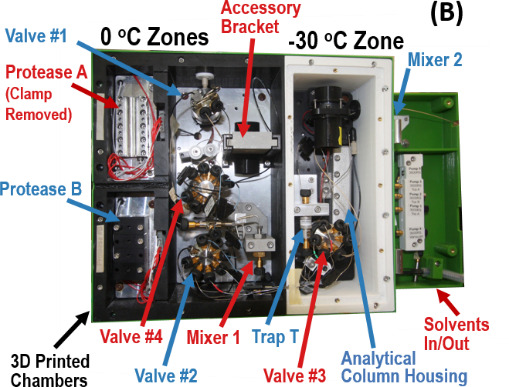

The system is integrated into a robotic rail system (Trajan Automation, Morrisville, NC)^2^2 Certain commercial materials and equipment are identified in order to adequately specify experimental procedures. Such identifications neither imply recommendation or endorsement by the National Institute of Standards and Technology nor does it imply that the material or equipment identified is the best available for the purpose. and accepts samples injected by a robotic syringe for processing through the proteolysis, desalting, and chromatographic separation steps of the HDX-MS measurement process. Figure 1 outlines the characteristics of this analysis platform. The apparatus includes an internal accessory bracket that can accommodate an electrochemical cell for the reduction of disulfide bonds. A twisted-pair network cable between the controller and computer allows annotation of chamber temperatures into the data records.

## 2 System Description

The subzero, dual protease column chromatography system comprises four, front-serviceable compartments, which are separately temperature-regulated to ±0.05 ^o^C. A removable, insulated bulkhead isolates the -30 ^o^C compartment, and a removable, insulated door seals the compartments and bulkhead from the ambient environment. The entire system is cooled with a liquid chiller (275 W @ -30 ^o^C) that circulates a 30% water/60% ethylene glycol/10% methanol mixture (v/v/v) through 0.95 cm OD stainless steel tubing that is clamped to the aluminum baseplate in each compartment. A throttle valve apportions the coolant flow between the 0 ^o^C (Zones 1, 3, 4) and -30 ^o^C (Zone 2) chambers. System cooling capacity is improved by employing a counter flow heat exchanger in the fluid return circuit of the 0 ^o^C coolant circuit.

Each coolant circuit has sufficient capacity to chill its aluminum plates below the setpoint. The temperature in each zone is reported to a process controller by thermistors and by resistance temperature detectors (RTD) that are screwed into the aluminum plates. To restore each plate to its desired temperature, a process controller circuit measures the appropriate sensor and applies a negative feedback algorithm to switch direct current (DC) to polyamide heaters until the setpoint temperature is restored. The control circuits regulate each aluminum zone to ± 0.05 ^o^C. To maintain uniform compartment air temperatures in Zones 1 and 2, mini-fans maintain convective air flows. To inhibit water condensation and frost, the box encasing the sample processing apparatus is sealed and purged with dry nitrogen. Two channels of the six-channel temperature controller proportionately regulate the temperatures of the LC valve extension shafts, preventing them from falling below the dew point temperature.

The fluidic circuit of the dual protease column chromatography system begins at an injection port on the top surface of the valve box in a location accessible to the robotic syringe arm. The fluidic system design assures good definition of temperature zone boundaries. At locations along the fluidic circuit where the chamber changes, capillaries are clamped to aluminum blocks, which are mounted on a compartment baseplate. The LC valves contact the aluminum compartment baseplates. Other fluidic components (e.g., Mixer 1, Trap T, Analytical column A) are encased in 3D-printed aluminum housings, which are mounted to a compartment baseplate. Contact with the thermal mass of each baseplate assures that fluidic components reside at the compartment setpoint temperature.

The apparatus has two chambers (Zones 2 and 3) that contain an immobilized protease column. Since the profile and dimensions of columns vary by vendor, each protease column is mounted in a 3D-printed aluminum or silver collet that fits the column profile exactly. The collet exterior presents a featureless, 8 mm diameter cylinder that is clamped into a matching holder. Each holder is affixed to a cooled, aluminum baseplate. A thermistor measures each clamp temperature, and the temperature controller maintains the clamp assembly at its setpoint temperature, typically (0 to 15) ^o^C ± 0.05 ^o^C.

Table 1 provides a general description of the LC pumps used in this apparatus. The original implementation of this system uses two Thermo Scientific UltiMate DGP-3600RS quaternary pumps, one Thermo Scientific Vanquish Binary Pump H (VH-P10-A), and one (optional) Thermo Scientific ISO-3100SD isocratic pump; however, any brand of pumps that is compatible with the (very adaptable) CHRONOS automation software (Trajan Scientific Americas Inc., Morrisville, NC) can be used. The valve states and flow rates of the chromatography pumps are controlled by CHRONOS.

**Table 1:** Pump functions, properties, and maximum operating pressures in the dual protease column HDX-MS analysis system.

Pump #	Function	Pump Type	Zone	Maximum Pressure (MPa)
1	Sample Loading	Quaternary	1	35
2	Protease Backflush Cleaning	Quaternary	2, 3	35
3	Adds ETG to prevent solvent freezing	Quaternary	1	35
4	Analytical Gradient	Binary	4	150
5 ^a^	Adds ACN to increase ESI efficiency	Isocratic		15

For chromatography the fluidic circuit requires a trap column (e.g., Phenomenex, Inc.; Torrance, CA; Model Kinetex EVO C18, 100 Å pore, 2.6 μm particle size, 20 mm long x 2.1 mm dia.) and an analytical column (e.g., Thermo Fisher Scientific; Waltham, MA; Model Hypersil GOLD C18, 100 Å pore, 1.9 μm particle size, 50 mm long x 1.0 mm dia.). Since solvent viscosities, particularly ETG/ACN/H2O mixtures, are high at -30 oC, the pressure capacity of Pump 4 largely governs choice of analytical column.

Quaternary pumps 1 and 2 supply aqueous solutions (99.9% H_2_O/0.1% formic acid (FA) v/v/v) for loading the sample into a protease column, for advancing peptides onto the trap column, and for backflushing and washing protease columns. (This mixture may change during cleaning cycles.) Pump 3 supplies 100% ETG to Mixer 1, which results in a 54.9% H_2_O/45% ETG/0.1% FA (v/v/v) solution. Binary Pump 4 provides the chromatography gradient, which is: Solvent A is 54.9% H_2_O/45% ETG/0.1% FA (v/v/v). Solvent B is 99.9% acetonitrile (ACN)/0.1% FA (v/v). Solvents A and B combine for a 50 µL/min flow rate. Typical gradient settings used are: 5% to 35% solvent B for 10 min, 35% to 60% solvent B for 30 min, 60% to 100% solvent B for 5 min, isocratic flow at 100% solvent B for 0.5 min, and a return in 5% solvent B for 0.5 min. Isocratic optional Pump 5 adds ACN to the analytical column effluent at the external Mixer 2, which can improve ESI source efficiency. Addition of a supercharging reagent to the solution at Pump 5 can increase the population of peptides in higher charge states, which is beneficial for HDX-MS experiments employing electron transfer dissociation methods [21]. The penalty of locating Valve #2 in the warmer Zone 2 is slight, as the additional exposure of sample to 0 ^o^C is ≈ 3 s. Its warmer location minimizes the cooling capacity required for stable operation at -30 ^o^C.

Figure 2 shows three primary operational states of the dual protease column, subzero, chromatography system. (Additional states exist.) In Fig. 2A the syringe fills the sample loop within Zone 1 (*T* ≈ 0 ^o^C), Pump 1 equilibrates Protease B, Pump 2 backflushes Protease A, and Pump 4 backflushes Trap T and Analytical Column A into the electrospray mass spectrometer (ESI-MS). As an option this system may be operated with a single protease column by replacing either Protease A or Protease B with a capillary. With this modification the system retains the facility for backflushing the protease column.

In Fig. 2B Pump 1 propels the protein sample through Protease B (*T* = (0 to 15) ^o^C). Protease B produces peptides that pass through Valve #4 into a Mixer 1, where Pump 3 enriches the ETG concentration to 45% ETG (v). The solution passes into Zone 2 (*T* = -30 ^o^C), where peptides become trapped on Trap T. Pump 2 backflushes Protease A. Pump 4 maintains Analytical Column A in a backflush cleaning cycle, which is monitored by the ESI-MS.

In Fig. 2C Pump 1 flushes the (now empty) sample loop through Protease A to waste, Pump 2 backflushes Protease B to waste, and Pump 4 passes a solvent gradient through Trap T and Analytical Column A, causing peptides to elute. The analytical column effluent passes through Valve #3 into Mixer 2, where ACN (and other additives) from Pump 5 (≈ 50 μL/min) increases the total flow to ≈ 100 μL/min. The lower viscosity mixture passes into the ESI-MS.

As is required of all chemical kinetic apparatus, system cleanliness is essential for good quality measurements. The selection of backflushing configurations enables removal of particulates from each column. While chromatographic gradients are in progress, it also possible to backflush either or both protease columns.

The design of this system allows the protease columns to remain in place throughout their life and for solvent to pass through them perpetually. Beyond assuring the availability of fresh protease columns, this arrangement saves start-up time by rendering unnecessary the customary, initial column conditioning process. The use of quaternary pumps allows protease column cleaning procedures to include gradients containing chaotropic agents and detergents. Finally, the availability of two columns containing different, pre-conditioned enzymes offers a convenient approach to increasing protein coverage by merging HDX-MS data from different enzymes.

**Fig. 2:** Subzero, dual protease column chromatography system shown in three operational states. For all cases Pump 3 adds ETG at Mixer 1 to solvent passing from Valve #4, and optional Pump 5 adds ACN at Mixer 2 to the effluent of Valve #3. **A)** Syringe fills the sample loop, Pump 1 equilibrates Protease B, Pump 2 backflushes Protease A, and Pump 4 backflushes Trap T and Analytical Column A into the ESI-MS. **B)** Pump 1 sweeps sample loop contents through Protease B and the proteolytic peptides are retained on Trap T (-30 ^o^C), Pump 2 backflushes Protease A, and Pump 4 backflushes Analytical Column A into the ESI-MS. **C)** Pump 1 flushes sample loop through Protease A to waste, Pump 2 backflushes Protease B to waste, and Pump 4 passes a solvent gradient through Trap T and Analytical Column A, causing peptides to elute into the ESI-MS. **Table:** 

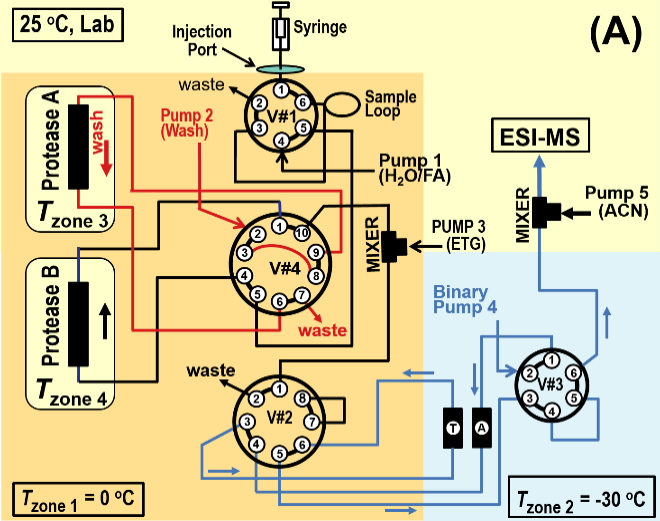	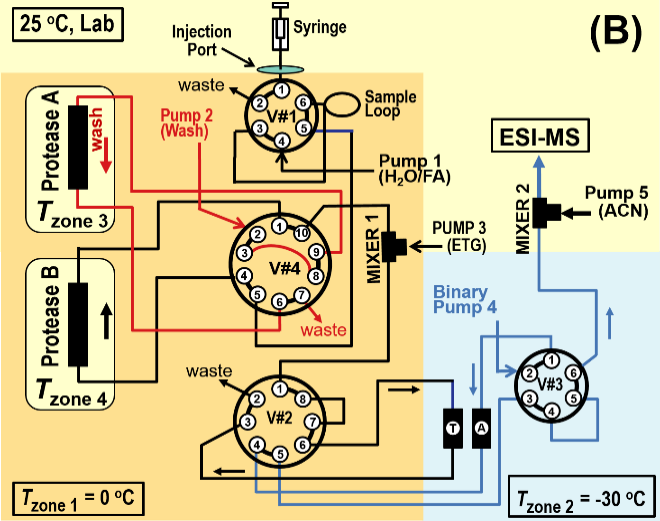
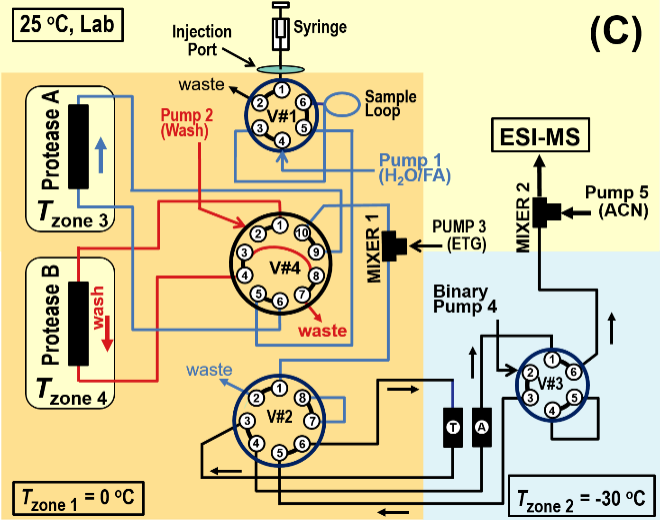

### 2.1 Audience

The components described herein are useful to the HDX-MS community. The multi-zone temperature controller may be used in any project that needs temperatures stabilized to ± 0.05 ^o^C over the range ± 30 ^o^C.

### 2.2 Materials

Materials are specified on the mechanical drawings of each component, which can be downloaded from the NIST Public Data Repository^3^3
https://doi.org/10.18434/M32151. The internal frame and compartment plates are aluminum (6061-T6). Delrin is used for the insulating standoffs. To suppress long-term corrosion from water, a corrosion-resistant sheet metal (1.60 mm, 16 gauge) is recommended for construction of the valve box housing and door. Interior compartment walls, much of the external housing, and the electrical component compartment that houses power supply and RTD components are 3D-printed in polylactide (PLA) plastic. The supplied STL files will support printing in several other plastics.

### 2.3 Tools or Equipment

The data resource containing schematics, mechanical drawings, and inventory of parts lists the specialized tools. It also contains component lists that specify most commercially-available electrical and mechanical components. Machine shop services are required to mill, drill, and tap the aluminum plates, Delrin spacers, and prefabricated boxes that house electronics. Sheet metal facilities are required to bend and weld the box and door. A shop must powder coat all sheet metal surfaces. A tube-bending service must prepare the coolant tubes that contact aluminum plates.

Many components are 3D printed. The housing may be printed by a commercial service. Alternately, it can be printed using any 3D printer having at least a 280 mm x 280 mm x 250 mm xyz work area. Parts 3D-printed in aluminum, bronze steel, or silver are best produced by a commercial service that can use the supplied STL files.

Assembly of components will require an adequate shop containing wrenches, blade and Phillips screw drivers, and Allen (hex) keys—both in English and metric sizes, a wire crimping tool, a tube cutting tool, and tube bender. A good quality soldering station is required for construction of the electrical circuits. Lead-free solder that contains flux is recommended.

### 2.4 Safety Considerations

Personal protective equipment (PPE) is required for some assembly procedures. Follow the manufacturer’s Safety Data Sheet for each material and chemical. Potentially lethal alternating current voltages (110 VAC) are present in the power supplies of the valve box, temperature controller, and valve control box. Only persons trained to safely work with such circuits should attempt to construct and test these circuits. The direct current voltages within the LC valve box are 24 VDC, which is considered well within safe voltage. However, contact with these circuits should be avoided. Whenever moving, testing, or opening a valve box or electrical chassis, remove all power by disconnecting the mains (110 VAC) source(s).

## 3 Instructions

Mechanical drawings of the LC valve system are presented in the same order necessary for final assembly. Throughout the document sub-assemblies and the location of sub-assemblies in the assembled apparatus are pictured periodically. The system requires capillaries that can withstand high-pressure (≈ 150 MPa, 1500 bar). For portions of the fluidic circuits peptide loss can be minimized by using PEEK or high-pressure capillaries with PEEK lining. After reviewing the recommendations of ref. [22], the end-user should specify these fittings and capillaries.
